# Health-promoting behaviour among adults in Germany – Results from GEDA 2019/2020-EHIS

**DOI:** 10.25646/8553

**Published:** 2021-09-15

**Authors:** Almut Richter, Anja Schienkiewitz, Anne Starker, Susanne Krug, Olga Domanska, Ronny Kuhnert, Julika Loss, Gert B. M. Mensink

**Affiliations:** Robert Koch Institute, Berlin Department of Epidemiology and Health Monitoring

**Keywords:** HEALTH-RELATED BEHAVIOUR, COMBINATIONS OF BEHAVIOUR, HEALTH-PROMOTING LIFESTYLE, ADULTS

## Abstract

Health-promoting behaviours are important at any age to prevent diseases and to promote well-being. Using data from GEDA 2019/2020-EHIS, a Germany-wide, representative survey, this article describes how often the adult population in Germany reports certain types of health-promoting behaviour in their everyday lives. The behaviours considered are nonsmoking, low-risk alcohol consumption, achievement of the World Health Organization’s (WHO) recommendations on aerobic physical activity, at least daily fruit and vegetable consumption, and maintaining a body weight within the normal range. This article describes the proportion of people who report these behaviours in their everyday lives by gender, age and education level, the number of health-promoting behaviours each person reports and the most common combinations in which they occur.

Young adults between 18 and 29 years are most likely to achieve a health-promoting lifestyle. The proportion of people who report at least 150 minutes of physical activity per week and a normal body weight is lower in later adulthood than among 18- to 29-year-olds. The recommendation to eat fruit and vegetables daily is implemented least often of all five aspects of health behaviour under study. Finally, women are more likely to lead a health-promoting lifestyle than men.

## 1. Introduction

Certain types of behaviour can help people maintain or improve their health. The COVID-19 pandemic demonstrated this with regard to infections: social distancing, appropriate implementation of hygiene rules on coughing and sneezing, as well as masks that cover mouth and nose have all been crucial in mitigating the spread of SARS-CoV-2. Just as there are measures that influence communicable diseases, certain forms of health-related behaviour play a significant role in the development or prevention of chronic diseases. An estimation for 2017 suggests that 11.6 million years of life were lost in Germany due to premature mortality [1]. Premature mortality refers to people dying at any age lower than their statistical life expectancy. Malignant neoplasms (35.2%) and cardiovascular diseases (27.6%) are the main causes of premature mortality in Germany [1]. Not smoking, low-risk alcohol consumption, regular physical activity, a healthy, plant-based diet and maintaining a body weight within the normal range can lower the risk of falling ill or dying prematurely from these conditions [2]. In particular, the interaction of several behaviours as part of an overall health-promoting lifestyle is associated with the greatest reduction in the risk of certain causes of death and overall mortality [3–8]. A meta-analysis with a mean observation period of 13.2 years found a combination of at least four health-promoting behaviours to be associated with a 66% reduction in all-cause mortality [7]. A study in the United States showed that women who reported five health-promoting behaviours could extend their lifespan after the age of 50 by 14.0 years and men could do so by 12.2 years compared with people who reported none of them [9].

**GEDA 2019/2020-EHIS**

Fifth follow-up survey of the German Health Update

**Data holder:** Robert Koch Institute

**Objectives:** Provision of reliable information on the health status, health behaviour and health care of the population living in Germany, with the possibility of European comparisons

**Study design:** Cross-sectional telephone survey

**Population:** German-speaking population aged 15 and older living in private households that can be reached via landline or mobile phone

**Sampling:** Random sample of landline and mobile telephone numbers (dual-frame method) from the ADM sampling system (Arbeitskreis Deutscher Markt- und Sozialforschungsinstitute e.V.)

**Sample size:** 23,001 respondents

**Study period:** April 2019 to September 2020

**GEDA survey waves:**

▶ GEDA 2009
▶ GEDA 2010
▶ GEDA 2012
▶ GEDA 2014/2015-EHIS
▶ GEDA 2019/2020-EHIS

Further information in German is available at www.geda-studie.de

According to a study based on data from the 2014 European Social Survey, only 5.8% of adults in Europe combine several forms of health-promoting behaviour, such as physical activity, not smoking, avoiding excessive levels of alcohol, eating fruit and vegetables every day and ensuring adequate sleep [10]. Similarly, the German Health Update (GEDA) 2009/2010 found that just 7.1% of women and 3.2% of men combined five forms of healthy behaviour. On the other hand, 29.1% of women and 17.8% of men reported to combine at least four out of five health-related behaviours [11].

Health-promoting lifestyles are determined not only by individual characteristics, but also by various social and economic as well as contextual factors. Moreover, different factors become effective at different ages, for example when social or family environment or time and financial resources change [12].

GEDA 2019/2020-EHIS provides current population-wide data that allow for a differentiated description of various health-related behaviours in Germany. The aim of this analysis is to determine the frequency of non-smoking, low-risk alcohol consumption, aerobic physical activity, daily consumption of fruit and vegetables, and normal-range body weight in Germany, and to identify any differences by gender, age and level of education. The health-related behaviours under study are considered individually and in different combinations.

## 2. Methodology

### 2.1 Study design and sample

GEDA is a nationwide cross-sectional survey of the German-speaking resident population in Germany. The GEDA study has been conducted by the Robert Koch Institute (RKI) on behalf of the German Federal Ministry of Health at multi-year intervals since 2008 and is part of the health monitoring at the RKI [13, 14]. The GEDA study analyses various topics such as health status, health behaviour, chronic diseases and the utilisation of health care services.

The fifth follow-up survey, GEDA 2019/2020-EHIS, took place between April 2019 and September 2020. As in the 2014/2015 wave, the questionnaire of the European Health Interview Survey (EHIS) was fully integrated [15, 16]. GEDA 2019/2020-EHIS was conducted as a telephone interview survey using a computer assisted, fully structured interview (i.e. Computer Assisted Telephone Interview, CATI). It was based on a random sample of landline and mobile telephone numbers (dual-frame method) [17]. The sample comprised the population aged 15 years and older living in private households and with permanent residency in Germany. A total of 23,001 people provided complete interviews for the GEDA 2019/2020-EHIS study. For the analyses set out here, these respondents were narrowed down to 22,708 people aged 18 or above. GEDA 2019/2020-EHIS used gender identities to describe gender differences and allowed the respondents to indicate which gender they felt they belonged to. Respondents 18 years and older included 11,959 women and 10,687 men. 62 respondents provided a different gender identity to the one that they were assigned at birth or gave no information at all. These individuals are not included in the gender stratified analyses.

Based on the standards of the American Association for Public Opinion Research (AAPOR), the response rate was 21.6% (RR3) [18].

A detailed description of the methodology applied for GEDA 2019/2020-EHIS can be found in Allen et al. in this issue of the Journal of Health Monitoring [19].

### 2.2 Indicators

Each of the health-promoting behaviours considered here is represented by a specific indicator.

#### Low-risk alcohol consumption

In GEDA 2019/2020-EHIS, the AUDIT-C (Alcohol Use Disorder Identification Test – Consumption Questions) was used to record the frequency and volume of alcohol consumption [20]. The participants were first asked about the frequency of their alcohol consumption in the last twelve months. Respondents who stated that they drank alcohol at least once a week were then asked about the number of standard drinks they consumed on weekdays (Monday to Thursday) and weekends (Friday to Sunday). This information was used to calculate the respondents’ mean consumption of pure alcohol per day in grams. In line with evidence-based guidelines [21, 22], ≤ 10 grams of pure alcohol per day for women and ≤ 20 grams per day for men was defined as low-risk level alcohol consumption. The indicator is considered fulfilled for those who stated that they had not drunk alcohol in the past twelve months, had done so less than once a month, or between once a month and two to three days per month and furthermore for people who reported that they had drunk alcohol at least once a week without exceeding the limits described above.

#### Current non-smoking

Data on smoking status was collected using the question: ‘Do you smoke tobacco products, including heated tobacco products?’ (Answer categories: ‘yes, daily’, ‘yes, occasionally’, ‘no, not any more’, ‘I have never smoked’). The answers were used to distinguish between current smoking (‘yes, daily’ or ‘yes, occasionally’) and current nonsmoking (‘no, not any more’ or ‘I have never smoked’). This article refers to the indicator ‘current non-smoking’.

#### Aerobic physical activity

The physical activity indicator was defined in line with the minimum recommendations for aerobic physical activity drawn up by the World Health Organization (WHO) [23, 24]. Data was gathered for the indicator using the German validated version of the European Health Interview Survey – Physical Activity Questionnaire (EHIS-PAQ) [25]. The participants were asked about their work-related, transport-related and leisure-time physical activity in a typical week. The indicator considers data on the weekly duration of at least moderate-intensity aerobic physical activity conducted during leisure time and the amount of time spent cycling used for transportation [25]. Data on walking was not included. Respondents undertaking aerobic physical activity for at least 150 minutes per week are considered to have fulfilled the conditions for the indicator.

#### Normal weight

Data on height and weight were reported by the respondents. Data on height was collected by asking: ‘How tall are you if you are not wearing shoes?’. The information was provided in centimetres. Data on body weight was collected with the question: ‘How much do you weigh if you are not wearing clothes and shoes? Please enter your weight in kg. Pregnant women should provide their weight before pregnancy’. Body mass index (BMI) is calculated using the ratio of body weight to height squared (kg/m^2^). The WHO classifies a weight within the normal range as a BMI between 18.5 kg/m^2^ and 25 kg/m^2^ [26].

Daily fruit and vegetable consumption The indicator ‘at least daily fruit and vegetable consumption’ was created to assess the consumption of fruit and vegetables. Data for the indicator was collected using the following questions: ‘How often do you eat fruit? Please include dried, frozen and canned fruit, but not fruit juices’. ‘How often do you eat vegetables and salads? Please include dried, frozen and canned vegetables, but not potatoes or vegetable juices’. Five response options were given in each case ranged from ‘daily or several times a day’ to ‘never’. Respondents who stated that they ate fruit and vegetables at least daily were categorised as ‘yes’ and fulfilled the conditions of the indicator. If one of the items was missing, the indicator variable was coded as missing.

#### Health-promoting lifestyle

The five indicators were used to assign an overall score to respondents’ health-promoting lifestyle. One point was awarded for each of the five behaviours reported if the corresponding indicator is realised. Lifestyles with a higher score can be viewed as healthier. In addition, a dichoto-mous variable is created using the total score (threshold value ≥4) to indicate that at least four of the five indicators were realised.

#### Sociodemography

The results are depicted by gender, age and education. The International Standard Classification for Education (ISCED) is used to classify the information provided by respondents on education [27]. The ISCED system takes into account both school and vocational qualifications and is particularly useful for international comparisons. ISCED categories 0 to 2 were grouped into a low, 3 to 4 into a medium, and 5 to 8 into a high education group.

### 2.3 Statistical analyses

The analyses are based on data from 11,959 women and 10,687 men aged between 18 and 99 years. For each indicator, respondents without information for the variables on which the indicator is based were excluded from the analyses. This led to the exclusion of 292 individuals for normal weight, 31 for fruit and vegetable consumption, 262 for aerobic physical activity, 314 for alcohol consumption and 9 for smoking. For the overall scores, respondents were excluded if they provided no data on one or more indicators (840 participants). Any categories representing less than 2% of respondents were aggregated with the next category, in order to mitigate the effect of the low number of cases and the lack of accuracy associated with such figures. The combinations of health-related behaviour are determined, and the most frequent combinations are presented.

The results for women and men are presented separately by age (18–29 years, 30–44 years, 45–64 years and ≥65 years) and education level (ISCED classification: low, medium, high education group). In order to test the independent influence on health-related behaviour of gender, age and education level, a logistic regression model was used that included these factors as influencing variables. The dichotomous variable mentioned above, which indicates whether the conditions for at least four of the five indicators were realised, was the outcome variable.

The analyses were carried out using a weighting factor to correct the sample for deviations from the population structure. For data weighting, design weighting was first applied to account for the different selection probabilities (of mobile and landline numbers). Subsequently, an adjustment based on the official population figures was carried out with regard to age, sex, federal state and district type (as of 31 December 2019). In addition, weighting also accounted for the distribution of education levels in the 2017 microcensus according to the ISCED classification [27].

The analyses were carried out using the procedures available in SAS 9.4. In order to take the weighting appropriately into account, confidence intervals (CI) and p-values were calculated using SAS survey procedures. A statistically significant difference between groups is assumed if the corresponding p-value in the Rao-Scott chi-square independence test is lower than 0.05.

## 3. Results

The following describes the percentage of the population that reported a health-related behaviour in their everyday life. The results are set out by women and men, age and education level (Table 1).

###  

#### Low risk alcohol consumption

**Figure 1:**
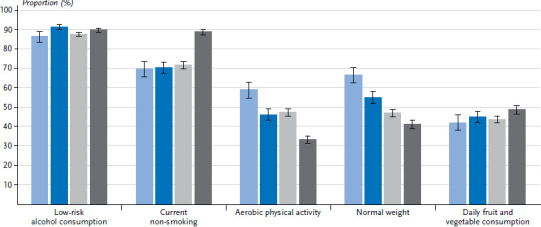
Percentage of women who realised the criteria for the particular indicators by age (n=11,959) Source: GEDA 2019/2020-EHIS

**Figure 2:**
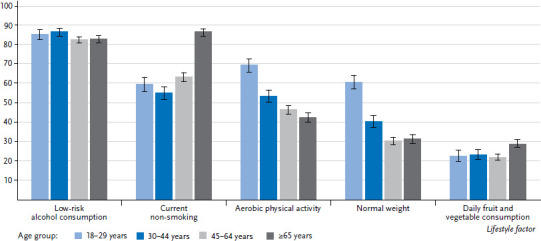
Percentage of men who realised the criteria for the particular indicators by age (n=10,687) Source: GEDA 2019/2020-EHIS

The vast majority of both women and men either do not drink alcohol, drink it rarely, or drink less than the respective amounts considered risky (Table 1). The highest percentage of low-risk alcohol consumption was found among women aged between 30 and 44, at 91.4% (Figure 1 and Figure 2). A larger proportion of women in the low education group reported low-risk alcohol consumption compared with women in the medium or high education group. This difference also exists between men, but is only statistically significant between the low and high education group.

#### Current non-smoking

76.0% of women and 66.1% of men do not currently smoke. The proportion of current non-smokers remains relatively stable up to the age of 65. However, it is significantly higher at retirement age than among younger aged groups. In addition, a positive association was identified between level of education and non-smoking; this trend is particularly pronounced among men. Slightly more than half of men in the low education group are current non-smokers, whereas almost 80% of men in the high education group do not currently smoke.

#### Aerobic physical activity

Overall, 44.8% of women and 51.2% of men meet the WHO recommendations on aerobic physical activity. The percentage decreases in both women and men with age, and is highest among 18- to 29-year-olds and lowest among people aged 65 or above. There is one exception: women between the ages of 45 and 64 meet the WHO’s recommendations on aerobic physical activity almost as often as women aged between 30 and 44. Women and men in the high education group achieve the recommendations more frequently than those in the medium and lower education group. In contrast to men, women in the medium education group also achieve the WHO’s recommendations on aerobic physical activity more often than those in the lower education group. The differences by education are more pronounced among women than among men.

#### Normal weight

Overall, 50.0% of women and 38.3% of men have a normal BMI. The percentage of women and men with a normal-range weight steadily decreases with age: whereas 66.6% of women and 60.7% of men aged 18 to 29 have a normal weight, the percentage falls to 41.1% among women and 31.4% among men aged 65 or above. In addition, women in the high education group are significantly more likely to have a normal weight than women in the low education group.

#### Daily fruit and vegetable consumption

Almost twice as many women (45.1%) as men (24.1%) reported that they ate fruit and vegetables every day. The proportion of people who do so remains relatively constant across age groups from young adulthood to the end of working life. After retirement, a few more women and men manage to integrate fruit and vegetables into their daily diets. Nevertheless, women continue to eat fruit and vegetables significantly more often every day compared with men. More people in the high education group eat fruit and vegetables every day compared with those in the medium or low education group. Women in the high education group fulfil the conditions for this indicator particularly often.

#### Health-promoting lifestyle

**Figure 3:**
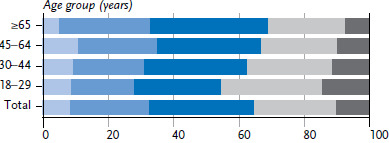
Achieved points of women in health-promoting lifestyle by age (n=11,469) Source: GEDA 2019/2020-EHIS

**Figure 4:**
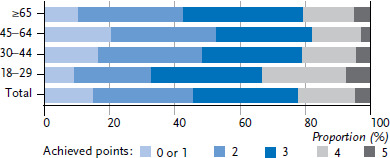
Achieved points of men in health-promoting lifestyle by age (n=10,337) Source: GEDA 2019/2020-EHIS

Most adults in Germany practice two or three health-promoting behaviours (56.2% of women and 62.5% of men). More women than men report four or five health-related behaviours at the same time (Figure 3, Figure 4 and Annex Table 1). Only one or none of these health-related behaviours is realised by 8.3% of women and 15.3%. Because only 1% to 2% of respondents reported none of the health-promoting behaviours under study, data on this category has been aggregated with the adjacent category.

In young adulthood, 45.8% of women and 33.4% of men report four or five health-related behaviours. This proportion reduces with age. The fact that every fifth men aged between 45 and 64 reported one or less achieved health-promoting behaviour is particularly striking; among women, it is only one in ten in this age group. On the other hand, in the group of 65-year-olds and older, the proportion of women and men with no more than one health-promoting behaviour is only about half that of the previous age group.

The results of the multivariate analyses show whether gender, age and education level are independently associated with health-promoting lifestyle. Women (OR=2.2; 95% CI 2.0–2.4) and people in the high (OR=2.8; 95% CI 2.4–3.4) or medium (OR=1.5; 95% CI 1.3–1.8) education group more frequently report at least four health-related behaviours. This also applies to people aged 18 to 29 (OR=2.0; 95% CI 1.7–2.3) compared with people aged 65 or above.

#### Most frequent combinations of health-related behaviours

In addition to the scores calculated for people’s health-promoting lifestyle, the combination of individual behaviours is also interesting. The most common combination identified among women is non-smoking together with low-risk alcohol consumption (Table 2). This is followed by a combination of all five indicators, and then the combination of non-smoking, low-risk drinking and daily fruit and vegetable indicators.

Similarly, the most common combination among men is non-smoking and low-risk alcohol consumption; however, this is followed by 13.5% of men who also achieve the WHO’s recommendations on aerobic physical activity during their everyday lives. A combination of all indicators, with the exception of daily fruit and vegetable consumption, is clearly less frequently.

## 4. Discussion

For a healthy lifestyle, it is recommended to eat a mostly plant-based and varied diet, to be physically active on a regular basis, to monitor one’s body weight, to drink alcohol in moderation and avoid smoking. Only a small proportion of the population fulfils all five of these health-promoting behaviours, with most adults in Germany achieving just two or three. A health-promoting lifestyle with four or five realised behaviours is most common among young adults aged between 18 and 29 years. Women are more likely to achieve a health-promoting lifestyle than men; people in the high education group are more likely to do so than those in the medium and lower education group.

In later adulthood the proportions of those who are physically active for at least 150 minutes per week or have a normal-range body weight are lower. The recommendation to eat fruit and vegetables daily is realised least often out of the five indicators under study. Men achieve the WHO recommendation on aerobic physical activity more often than women.

Few studies that use data from a population-based sample describe health-related behaviour in a similar way. These studies also vary in the number and type of behaviour parameters that they analyse and the criteria that they use to do so. However, similar to the present results, it can be seen consistently that only a minor proportion of the adult population fulfils the conditions for all of the indicators that were studied; this not only applies in the case of an earlier study in Germany [11] but also across Europe [10] and even worldwide [28–31].

More women than men adopt a lifestyle that is in line with the recommendations on health-related behaviour [11, 29–33], but other surveys have also found that men fulfil the recommendations for physical activity more often compared with women [11, 31]. Other studies have also identified better health-related behaviour among higher educated groups [29–31, 34]. The association between age and health-related behaviour is less clear, depending on study and country, different age groups had a better health-related behaviour [10, 29, 31, 35].

A meta-analysis examined whether certain clusters of health-promoting behaviours occur more frequently together, and whether their prevalence differs between subgroup [36]. The most common cluster identified by the analyses set out here was that of non-smoking combined with low-risk alcohol consumption; this applies to both women and men. The same combination has been identified by other studies [36]. The analyses set out here only rarely found a combination of a healthy diet – assessed as daily fruit and vegetable consumption – and regular physical activity, despite the fact that addressing both factors is recommended to prevent overweight [37, 38]. However, in the present study, dietary behaviour is represented in a simplified way which may explain the lower correlation identified here with physical activity and BMI.

Both the number of reported health-related behaviours and the way in which they are combined are important for disease prevention [6, 39, 40]. In particular, combinations that include smoking are associated with an increased risk of all-cause mortality and mortality from cardiovascular diseases [6]. In contrast, people that combine a normal weight and at least two of the factors non-smoking, moderate alcohol consumption and physical activity were found to have a particularly high number of years of life free from non-communicable diseases (such as diabetes mellitus and cardiovascular diseases) [39].

GEDA 2019/2020-EHIS is based on a large sample which allows representative statements for population health in Germany [19]. GEDA was conducted with a high degree of standardisation. One limitation of the study, however, is its survey mode. The use of self-reported data instead of measurements can influence results, for example of body height and weight, as people tend to underestimate their weight and overestimate their height [41]. The analyses undertaken here only examined a selection of behaviours that are considered to promote health. The available data made it impossible to consider factors such as sleep behaviour or how a person deals with stress. In line with previous studies, maintaining a normal weight was regarded as an independent health-promoting behaviour [7–9, 11, 29–31]. However, it can also be viewed as resulting from the interplay of diet and exercise. Ultimately, there is no standard established definition of a healthy lifestyle, and different operationalisations are used in different studies [10, 11, 28–30].

This study describes a health-promoting lifestyle in terms of a simple overall score. This values each health-related behaviour equally, even though the preventive importance of a particular behaviour can be different and vary depending on the target variable (risk associated with certain diseases, disease-specific mortality or all-cause mortality). With a score of two it cannot be assumed that people generally live a healthy lifestyle (they fail to fulfil the conditions of three other indicators). For a lifestyle with at least four health-relevant behaviours many studies have shown health benefits [7].

The indicators used are based on the questionnaire of the European Health Interview Survey (EHIS) [15, 16]. The reference periods differ depending on the indicator (e.g. ‘in the last year’ for alcohol consumption or ‘current’ for smoking). This article assessed health-related behaviour using recommendations and threshold values. However, the selected threshold values oversimplify the respective behaviour. For example, if both aspects of the WHO’s recommendations on physical activity, i.e. aerobic physical activity and muscle strengthening (on at least two days per week) [23, 24], were used, only 23.1% of women and 29.0% of men would fulfil the conditions for this indicator. At the same time, the selected threshold values do not allow to differentiate in more detail: for example, respondents who do not consume fruit on a daily basis do not meet the conditions for this indicator, although they may eat a large amount of vegetables. This demonstrates that improvements to individual health-related behaviour that are below the selected threshold values are also desirable and can come with health benefits. It has also been argued that people should completely avoid alcohol in order to reduce overall mortality [42]. In addition, the behaviours considered here could be assessed in a more differentiated manner, for example nutrition could have been analysed by focusing on other types of food. However, not enough data were available to do so.

Further information on the GEDA study is described in detail elsewhere [19]. Part of the data collection by GEDA 2019/2020-EHIS was conducted at the beginning of the COVID-19 pandemic. From mid-March 2020, extensive containment measures and policies came into effect, such as contact restrictions, and the closure of schools, shops, restaurants, and many public facilities. Initial evaluations of the possible impact of these measures on health behaviour have identified a higher body weight and a higher BMI among the population compared with the same period in 2019. In contrast, the number of tobacco smokers has decreased [43]. The COVID-19 pandemic containment measures, therefore, appear to have resulted in changes in individual behaviour. However, the impact that these measures will have on health at the individual and population level remains to be seen [44].

The results demonstrate that health-related behaviours can differ significantly by age and gender. This underscores the need for preventive and health-promoting measures that take into account people’s heterogeneous needs, requirements and circumstances. Men, for example, consume less often fruit and vegetables on at least a daily basis; whereas women are less likely to follow the recommendations on physical activity than men. This provides a starting point for measures that take these gender-based differences into account. However, such measures can only be effective if they also account for the specific causes and barriers to health-promoting or risky behaviour that apply in each case. These include issues such as gender roles and social constructs of femininity and masculinity [45]. This not only applies when addressing these issues [46, 47], but also for context-related interventions and structural changes that may result in different possibilities for women and men. So far there has been little research on gender-sensitive approaches to preventive measures, especially with regard to how these measures can contribute towards breaking down gender stereotypes instead of consolidating them (gender-transformative prevention) [48, 49].

Differences in health behaviour have also been identified by age. The proportion of non-smokers increases with age and is highest among people aged 65 or above. In contrast, achievement of the WHO’s recommendations on aerobic physical activity decreases with age. There are many possible explanations for these differences. For example, the decline in physical fitness and the increase in frailty, especially among over 65-year-olds, could explain the age-related differences in physical activity. In addition to biological aspects, however, social aspects can also play a role. There are signs that certain biographical events and changes in people’s lives can have an impact on health-related behaviour and BMI, for example, retirement and changes in family status (starting a family, divorce, losing a partner, children leaving the family home) [50–53]. The particularly favourable health-related behaviour identified among people aged 65 or above (e.g. not smoking) could also be influenced by selection effects, as the proportion of people with high-risk behaviour may be lower in older age groups due their possibly shorter life expectancy.

However, in general, neither age nor gender alone can explain the difference in the prevalence of health-relevant behaviour. Socioeconomic factors also play an important role. Across all age groups and among all of the behaviours considered, favourable health-related behaviour is found significantly more often among people with a higher level of education compared with people with a lower level of education. This association has already been described in the literature [34] and underscores the need for preventive and health-promoting measures to be planned in a manner that particularly provides health-related options for people with a low level of education. Low-risk alcohol consumption is an exception: women in the low education group are more likely to demonstrate low-risk alcohol consumption than women in the high education group. This is confirmed by other national and international studies [54, 55]. One of the explanations discussed in the literature is that different role models exist: women with a higher education level tend to face greater professional demands and earn a higher income, aspects that are linked to traditional ‘male’ roles, which is then further reflected in their risky alcohol consumption [56]. However, these findings can also be explained against the background of changing cultural and social norms, such as to women’s position in society [57]. More research is needed into consumption patterns, alcohol products that women consume and how and when they are drinking alcohol. Furthermore, this research would need to be conducted against the background of gender-based differences and differences in social circumstances. Finally, more research is needed into how measures can contribute towards achieving health equity.

Differences between population groups arise not only at the level of individual health-related behaviour, but also at the lifestyle level (when considered as the overall score of the five selected health behaviours). The results show that the highest percentage of people who combine four or five of the behaviours under study can be found in the youngest study group – people aged 18 to 29. However, this still only applies to 33.0% of men in this age group; and it does apply to significantly more women, at 45.3%. The overall score provides a useful means of assessing the preventive relevance of a particular lifestyle. The more beneficial behaviours are combined, the more likely it is to have a decreased risk of morbidity from various chronic diseases [8].

Instead of fostering health-promoting behaviour, the given societal framework and context factors often hinder health-promoting behaviour. As long as healthy choices are not the easiest ones to make [58], people will find it difficult to regularly fulfil the requirements for all health-promoting indicators in their everyday lives. Appropriate measures must therefore not only promote individual health behaviours (e.g. only exercise), but create the conditions that people need in order to realise their greatest possible health potential in different areas. Moreover, it is also important to mention here that although improved health-related behaviour is beneficial at the individual level, a plant-based diet with plenty of fruit and vegetables and increased physical activity through walking and cycling instead of using motorised transport can also contribute to protecting the climate [59].

This study demonstrates the need for measures that encourage people to develop and maintain behaviours that are beneficial to their health in their everyday life beyond young adulthood. Overall, the data suggest that certain health-promoting and certain risky behaviours can occur together. Therefore, approaches are needed that account for the interactive nature of various health-related behaviours. Effective approaches are required that enable people to change multiple health-related behaviours. Moreover, these approaches also need to be gender-sensitive and to be conceived and made particularly accessible to socially disadvantaged groups.

## Key statements

- A health-promoting lifestyle includes behaviours such as not smoking, low-risk alcohol consumption, daily fruit and vegetable consumption, regular physical activity following international recommendations, and maintenance of a body weight within the normal range.
- Significantly more women than men practice at least four out of five health-promoting behaviours (35.6% of women and 22.1% of men).
- Most adults in Germany report implementing two or three out of five health-promoting behaviours (56.2% of women and 62.5% of men).
- Young adults are more likely to achieve a health-promoting lifestyle than people in the older age groups.
- Women and men in the higher education group are more likely to achieve a health-promoting lifestyle than people in the medium and low education group.

## Figures and Tables

**Table 1:** Health-related behaviour by gender, age and education level (n=11,959 women, n=10,687 men) Source: GEDA 2019/2020-EHIS

	Low-risk alcohol consumption^1^		Current non-smoking		Aerobic physical activity^2^		Normal weight^3^		Daily fruit and veg etable consumption	
	%	(95% CI)	%	(95% CI)	%	(95% CI)	%	(95% CI)	%	(95% CI)
**Total**										
Women	88.9	(88.1–89.7)	76.0	(74.7–77.3)	44.8	(43.5–46.1)	50.0	(48.6–51.4)	45.1	(43.8–46.5)
Men	83.9	(82.8–85.0)	66.1	(64.6–67.5)	51.2	(49.8–52.7)	38.3	(36.9–39.7)	24.1	(22.9–25.3)
p-value^4^		<0.0001		<0.0001		<0.0001		<0.0001		<0.0001
**Age group**										
**Women**										
18–29 years	86.4	(83.2–89.1)	69.6	(65.4–73.6)	58.9	(54.6–63.0)	66.6	(62.4–70.6)	42.1	(38.0–46.3)
30–44 years	91.4	(89.8–92.8)	70.4	(67.2–73.4)	46.2	(43.1–49.4)	55.1	(51.9–58.3)	44.9	(41.8–48.0)
45–64 years	87.6	(86.3–88.8)	71.8	(69.7–73.7)	47.3	(45.3–49.3)	47.0	(44.9–49.0)	43.7	(41.7–45.7)
≥65 years	89.8	(88.5–91.0)	88.7	(87.1–90.2)	33.3	(31.2–35.4)	41.1	(38.8–43.5)	48.7	(46.3–51.1)
p-value^4^		0.0006		<0.0001		<0.0001		<0.0001		0.0108
**Men**										
18–29 years	85.2	(82.2–87.8)	59.5	(55.7–63.3)	69.3	(65.6–72.7)	60.7	(56.9–64.4)	22.7	(19.8–25.8)
30–44 years	86.4	(83.9–88.5)	55.0	(51.6–58.3)	53.5	(50.2–56.8)	40.4	(37.2–43.6)	23.4	(20.8–26.1)
45–64 years	82.4	(80.5–84.2)	63.3	(60.9–65.6)	46.4	(44.1–48.7)	30.4	(28.3–32.5)	22.0	(20.2–24.0)
≥65 years	82.7	(80.6–84.7)	86.4	(84.2–88.3)	42.6	(40.0–45.2)	31.4	(29.0–33.9)	28.9	(26.7–31.3)
p-value^4^		0.0350		<0.0001		<0.0001		<0.0001		0.0002
**Education level**										
**Women**										
Low education group	92.9	(90.7–94.7)	72.4	(68.6–76.0)	27.4	(24.0–31.0)	42.7	(38.7–46.8)	42.3	(38.4–46.3)
Medium education group	89.1	(88.0–90.1)	74.7	(73.1–76.2)	46.7	(45.0–48.4)	48.8	(47.1–50.5)	42.9	(41.2–44.6)
High education group	84.1	(82.6–85.4)	83.3	(81.7–84.7)	56.6	(54.7–58.4)	60.4	(58.5–62.1)	54.6	(52.7–56.4)
p-value^4^		<0.0001		<0.0001		<0.0001		<0.0001		<0.0001
**Men**										
Low education group	88.3	(84.2–91.4)	54.9	(49.5–60.2)	46.0	(40.7–51.4)	35.5	(30.7–40.7)	23.1	(19.0–27.8)
Medium education group	83.7	(82.0–85.2)	62.5	(60.4–64.5)	49.3	(47.3–51.4)	37.6	(35.6–39.6)	21.2	(19.6–22.8)
High education group	82.2	(80.9–83.4)	78.0	(76.6–79.4)	57.3	(55.7–58.8)	40.8	(39.2–42.4)	29.9	(28.5–31.3)
p-value^4^		0.0108		<0.0001		<0.0001		0.0715		<0.0001

CI=confidence interval

^1^ Mean consumption of ≤10 grams of pure alcohol per day for women and ≤20 grams per day for men

^2^ Achievement of the World Health Organization’s (WHO) recommendations on aerobic physical activity (at least 150 minutes per week)

^3^ In line with the standards used by the WHO, a body mass index ranging from 18.5kg/m^2^ to 25kg/m^2^

^4^ Rao-Scott chi-square independence test

**Table 2:** Proportions of the 15 most common combinations of health-related behaviours by gender (n=11,469 women, n=10,337 men) Source: GEDA 2019/2020-EHIS

Number assigned to the combination	Low-risk alcohol consumption^1^	Current non-smoking	Aerobic physical activity^2^	Normal weight^3^	Daily fruit and vegetable consumption	%
**Women**						
**1**	+	+	–	–	–	12.4
**2**	+	+	+	+	+	10.5
**3**	+	+	–	–	+	9.4
**4**	+	+	–	+	–	8.1
**5**	+	+	+	+	–	7.9
**6**	+	+	+	–	+	6.9
**7**	+	+	–	+	+	6.8
**8**	+	+	+	–	–	6.3
**9**	+	–	–	–	–	4.7
**10**	+	–	–	+	–	4.2
**11**	+	–	+	+	–	2.5
**12**	+	–	–	–	+	2.0
**13**	+	–	+	–	–	2.0
**14**	+	–	+	+	+	1.9
**15**	+	–	–	+	+	1.7
**Men**						
**1**	+	+	–	–	–	13.9
**2**	+	+	+	–	–	13.5
**3**	+	+	+	+	–	8.7
**4**	+	–	–	–	–	7.4
**5**	+	+	–	+	–	5.6
**6**	+	+	+	–	+	5.1
**7**	+	–	+	+	–	4.7
**8**	+	+	+	+	+	4.7
**9**	+	–	+	–	–	4.6
**10**	+	+	–	–	+	4.4
**11**	+	–	–	+	–	4.4
**12**	–	+	–	–	–	2.2
**13**	–	+	+	–	–	2.1
**14**	–	–	–	–	–	2.0
**15**	+	+	–	+	+	1.9

+ health-promoting behaviour was reported, - health-promoting behaviour was not reported

^1^ Mean consumption of ≤10 grams of pure alcohol per day for women and ≤20 grams per day for men

^2^ Achievement of the World Health Organization’s (WHO) recommendations on aerobic physical activity (at least 150 minutes per week)

^3^ In line with the standards used by the WHO, a body mass index ranging from 18.5kg/m^2^ to 25kg/m^2^

## Appendix

**Annex Table 1:** Proportion of achieved points in health-promoting lifestyle by gender and age (n=11.469 women, n=10.337 men) Source: GEDA 2019/2020-EHIS

Points	Age group				
	18–29 years	30–44 years	45–64 years	≥65 years	Total
**Women**					
**0 or 1** **2** **3** **4** **5**	8.3% 24.0% 32.2% 25.1% 10.5%	8.6% 19.3% 26.4% 31.0% 14.8%	9.0% 21.8% 31.7% 25.8% 11.7%	10.6% 24.2% 31.7% 23.5% 10.0%	4.7% 28.0% 36.2% 23.4% 7.7%
**Men**					
**0 or 1** **2** **3** **4** **5**	15.3% 30.3% 32.2% 17.4% 4.7%	9.2% 23.3% 34.1% 25.7% 7.7%	16.8% 31.4% 30.6% 16.7% 4.6%	20.6% 32.1% 29.4% 14.9% 3.0%	10.4% 31.9% 37.0% 15.5% 5.2%
