# Erratum: Health-promoting behaviour among adults in Germany – Results from GEDA 2019/2020-EHIS

**DOI:** 10.25646/9853

**Published:** 2022-03-30

**Authors:** Richter A, Schienkiewitz A, Starker A, Krug S, Domanska O

Richter A, Schienkiewitz A, Starker A, Krug S, Domanska O et al. (2021)

Journal of Health Monitoring 6(3): 26–44.

DOI 10.25646/8553

In the ‘Annex Table 1’ table on page 43, the naming of the five results columns (four age groups plus total) was inadvertently shifted, resulting in incorrect headings above the correct percentages.

The article has been corrected.

The corrected version of the article is available at DOI 10.25646/8553.2

